# Microtubules Regulate Migratory Polarity through Rho/ROCK Signaling in T Cells

**DOI:** 10.1371/journal.pone.0008774

**Published:** 2010-01-19

**Authors:** Aya Takesono, Sarah J. Heasman, Beata Wojciak-Stothard, Ritu Garg, Anne J. Ridley

**Affiliations:** 1 University College London, Department of Biochemistry and Molecular Biology and Ludwig Institute for Cancer Research, London, United Kingdom; 2 School of Biosciences, University of Exeter, Exeter, United Kingdom; 3 Randall Division of Cell and Molecular Biophysics, King's College London, London, United Kingdom; 4 Department of Experimental Medicine and Toxicology, Imperial College London, London, United Kingdom; University of Birmingham, United Kingdom

## Abstract

**Background:**

Migrating leukocytes normally have a polarized morphology with an actin-rich lamellipodium at the front and a uropod at the rear. Microtubules (MTs) are required for persistent migration and chemotaxis, but how they affect cell polarity is not known.

**Methodology/Principal Findings:**

Here we report that T cells treated with nocodazole to disrupt MTs are unable to form a stable uropod or lamellipodium, and instead often move by membrane blebbing with reduced migratory persistence. However, uropod-localized receptors and ezrin/radixin/moesin proteins still cluster in nocodazole-treated cells, indicating that MTs are required specifically for uropod stability. Nocodazole stimulates RhoA activity, and inhibition of the RhoA target ROCK allows nocodazole-treated cells to re-establish lamellipodia and uropods and persistent migratory polarity. ROCK inhibition decreases nocodazole-induced membrane blebbing and stabilizes MTs. The myosin inhibitor blebbistatin also stabilizes MTs, indicating that RhoA/ROCK act through myosin II to destabilize MTs.

**Conclusions/Significance:**

Our results indicate that RhoA/ROCK signaling normally contributes to migration by affecting both actomyosin contractility and MT stability. We propose that regulation of MT stability and RhoA/ROCK activity is a mechanism to alter T-cell migratory behavior from lamellipodium-based persistent migration to bleb-based migration with frequent turning.

## Introduction

Cell migration is essential for the recruitment of T cells to and circulation within lymphoid organs, where they encounter antigen-presenting dendritic cells, and in tissues during immune surveillance, immune responses and inflammation. Migrating T cells are normally morphologically polarized with spatially distinct front (lamellipodium) and rear (uropod) structures, and migrate by extending the lamellipodium forwards and retracting the uropod [1]–[3]. In lymph nodes in vivo, T cells migrate rapidly and for many hours until they encounter antigen [4]. In vitro, T cells polarize spontaneously, for example on the integrin ligand ICAM-1 [3], and this requires activation of the integrin LFA-1 [5]. Similarly, neutrophils polarize and migrate in a uniform concentration of chemokine [6], [7], a process that has been termed “self-organizing polarity” [8], [9].

Cell polarization and migration require dynamic rearrangement of the actin and microtubule cytoskeletons via intracellular signaling pathways involving Rho family GTPases [10]–[12]. Lamellipodium extension in T cells requires Rac-induced actin polymerization [13], whereas the uropod is enriched in cell adhesion molecules such as ICAM-3 and CD44 that associate with ezrin/radixin/moesin (ERM) proteins, which in turn link these receptors with the cortical actin cytoskeleton [14]. Rho signaling is required for uropod extension and for detachment of the rear of migrating T cells [3], [15]. Rho is also well known to stimulate myosin light chain (MLC) phosphorylation and hence actomyosin contractility [16].

Disruption of MTs by MT depolymerizing agents such as nocodazole affects cell polarity and directional lamellipodium extension in several cell types, including neutrophils [7],[17]–[21], but the effects of MT depolymerization on T cell migration have not been studied in detail [22], [23]. In migrating T cells, the microtubule-organizing center (MTOC) is positioned behind the nucleus and MTs are predominantly localized in the uropod, which has been proposed to facilitate deformability of T cells [23]. In contrast, MTs and the MTOC polarize towards an antigen-presenting cell during formation of an immune synapse [13].

Rho GTPases both regulate and are regulated by MT dynamics. For example, MT depolymerization by nocodazole has been shown to activate RhoA, in part through release of the MT-associated RhoGEF, GEF-H1 [24], [25]. On the other hand, RhoA acts via its target mDia to mediate lysophosphatidic acid-induced MT stabilization at the edge of a scratch wound in fibroblasts [26], [27], and appears to inhibit MT dynamics [28]. Rac1 promotes microtubule growth at the leading edge of migrating cells through its target PAK1, which phosphorylates and inhibits the MT-destabilising protein Op18/stathmin [29].

Here, we investigate the inter-relationship between MTs and Rho signaling in T cell migration. We find that MT disruption results in frequent turning of cells during migration, reflecting loss of a stable uropod structure and increased membrane blebbing. Inhibition of ROCK serine/threonine kinases, which are RhoA targets, increases MT stability, inhibits blebbing and restores migratory polarity, indicating that RhoA/ROCK signaling regulates both contractility and MT dynamics during migration.

## Results

### Microtubules Are Required for Migratory Persistence of T Cells

**Figure 1:**
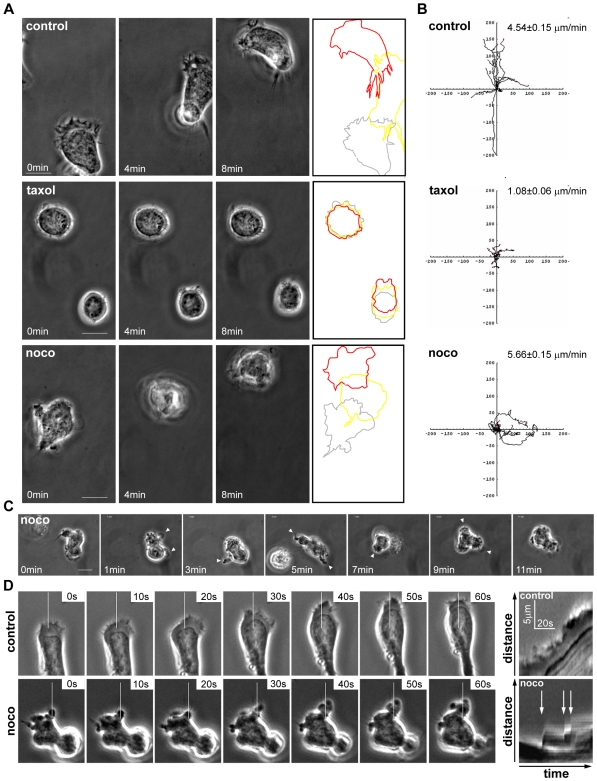
Microtubule dynamics are required for stable T cell migratory polarity. CCRF-CEM T cells were pre-treated with or without 10 µM taxol for 30 min or 20 µM nocodazole for 10 min, plated on ICAM-1-coated dishes, and stimulated with 1 nM CXCL12. Cell migration was monitored by time-lapse microscopy. (A) Frames from movies of control, taxol or nocodazole-treated CCRF-CEM cells at 0, 4 and 8 min are shown. Outlines of the cells at 0 (gray), 4 (yellow) and 8 (red) min reveal the migrating paths. Bars = 10 µm. (B) Migration tracks of cells (10 to 16 cells for each condition) are displayed as direction plots. The mean migration speed of cells ± SEM is shown. Data shown are representative of 4 independent experiments. (C) Frames of a nocodazole-treated CCRF-CEM cell (Movie S8). White arrowheads indicate bleb-like protrusions. (D) Frames of a control (top panels; Movie S4) and nocodazole-treated (bottom panels; Movie S5) CCRF-CEM cell are shown. Right panels show kymographs of the region marked by the white bar in the left panels. White arrows (bottom panel) indicate extension of progressive blebs. Bar = 10 µm.

Stimulation of CCRF-CEM T cells with the chemokine CXCL12/SDF-1α rapidly induced migratory cell polarity, with a lamellipodium at the front and uropod at the back, and migration on ICAM-1 (Figure 1A, B, Movie S1). To study the contribution of MTs to T cell polarization and migration, cells were treated with MT stabilizing (taxol) or depolymerizing (nocodazole) reagents. Taxol prevented morphological polarization and migration on ICAM-1, and the majority of cells remained stationary with a spherical morphology (Figure 1A, B, Movie S2). In contrast, nocodazole did not significantly affect migration speed, but caused unstable polarity and frequent turning (Figure 1A, B, Movie S3). Most nocodazole-treated cells also did not form a stable uropod structure which could dictate the axis of migration. Instead, small uropod-like protrusions formed only transiently and then retracted (Movie S3). The microtubule-depolymerizing agent colchicine similarly induced loss of T cell lamellipodial/uropod migratory polarity (data not shown).

High-resolution analysis by timelapse microscopy indicated that nocodazole-treated cells often extended blebbing membrane protrusions rather than lamellipodia (Figure 1C, D, Movies S4 and S5). Sequential membrane blebs could extend in one region of the plasma membrane, allowing the cell to move progressively in one direction, accompanied by cytoplasmic flow towards the blebbing area (Figure 1D, Movie S5). Similar membrane blebbing was also observed in nocodazole-treated human T-lymphoblasts (Movies S6 and S7), indicating that this response is not restricted to CCRF-CEM cells.

### Microtubules Regulate T Cell Migratory Polarity

**Figure 2:**
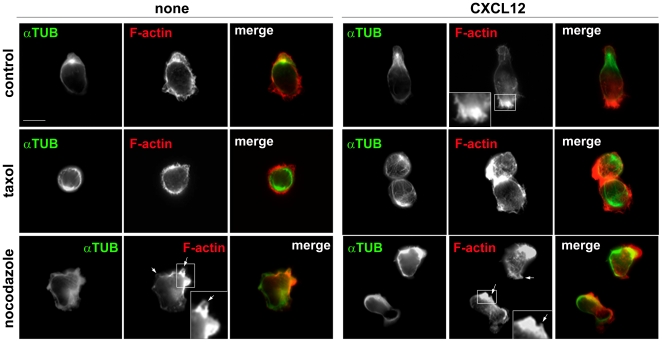
Microtubule dynamics are required for polarized distribution of F-actin. CCRF-CEM cells were pre-treated with or without taxol or nocodazole, plated on ICAM-1, and then stimulated with 20 nM CXCL12 for 5 min. Cells were fixed and stained with anti-α-tubulin antibody (green) and TRITC-conjugated phalloidin to show actin filaments (red). Representative confocal images are shown. Bar = 10 µm. The white arrows in nocodazole-treated cells indicate bleb-like membrane protrusions.

**Figure 3:**
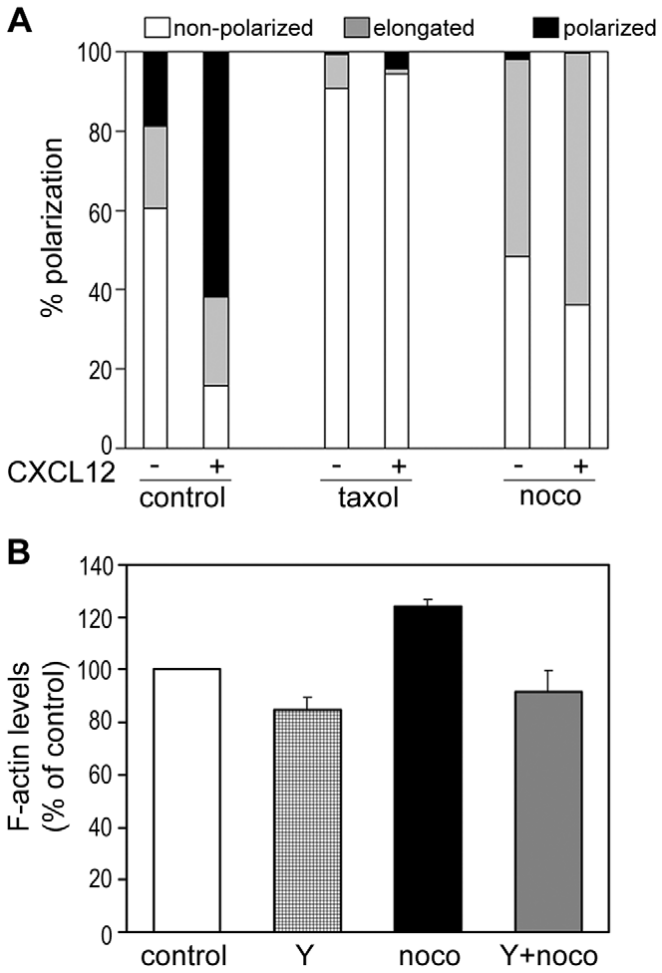
Effects of microtubule-disrupting agents on T-cell polarization and F-actin levels. (A) Quantification of T cell polarization. T cell morphology was classified in 3 categories: 1) non-polarized (white), where the cells had a spherical morphology, 2) elongated (gray), where the cells had an elongated cell shape but no diametric polarization of F-actin and α-tubulin, and 3) migratory polarized (black), where the cells were elongated and had diametric distribution of F-actin at the leading edge and the MTOC (identified with anti-α-tubulin antibody) behind the nucleus; n = 110 to 150 cells for each condition from 3 independent experiments. (B) Nocodazole alters F-actin levels in T cells. Flow cytometric analysis of the F-actin content of CCRF-CEM cells incubated with 10 µM Y-27632 for 30 min and/or 20 µM nocodazole for 10 min. Data are shown as a percentage of the mean fluorescence of untreated cells. Data are the mean of three independent experiments +/− SEM. *p<0.05 compared to control cells.

The effects of taxol and nocodazole on T cell migratory polarity were quantified by analysing F-actin and α-tubulin distribution. Prior to CXCL12 stimulation, CCRF-CEM cells did not have a polarized distribution of F-actin, but CXCL12 rapidly induced F-actin accumulation at one side of the cell, representing a single lamellipodium, and cell elongation (Figure 2, Figure 3A). Most MTs and the microtubule-organizing centre (MTOC) were located behind the nucleus in the uropod of migrating CCRF-CEM T cells, consistent with previous studies in neutrophils and T cells (Figure 2) [7], [20], [23], [30], [31]. CXCL12-stimulated cell elongation and polarization was inhibited by taxol (Figure 2, Figure 3A), although lamellipodia and membrane ruffles were often observed around the circumference of taxol-treated cells in response to CXCL12 (Figure 2). Nocodazole-treated cells were frequently elongated but did not show the characteristic migratory polarity of T cells (Figure 2, Figure 3A). Instead, F-actin often accumulated in multiple small bleb-like protrusions in both unstimulated and CXCL12-stimulated cells (Figure 2, nocodazole-treated cells, indicated with arrows), in agreement with the membrane blebbing observed by timelapse microscopy (Figure 1C, Movie S5). Recently, it has been reported that F-actin accumulates in membrane blebs as they start to retract [32], and thus it is likely that these F-actin-enriched protrusions represent retracting blebs. F-actin levels were increased in nocodazole-treated T cells (Figure 3B), similar to observations that nocodazole increases F-actin levels in neutrophils [20] and induces stress fibers in fibroblasts [33].

### Nocodazole Does Not Prevent Polarized Distribution of Uropod Proteins

**Figure 4:**
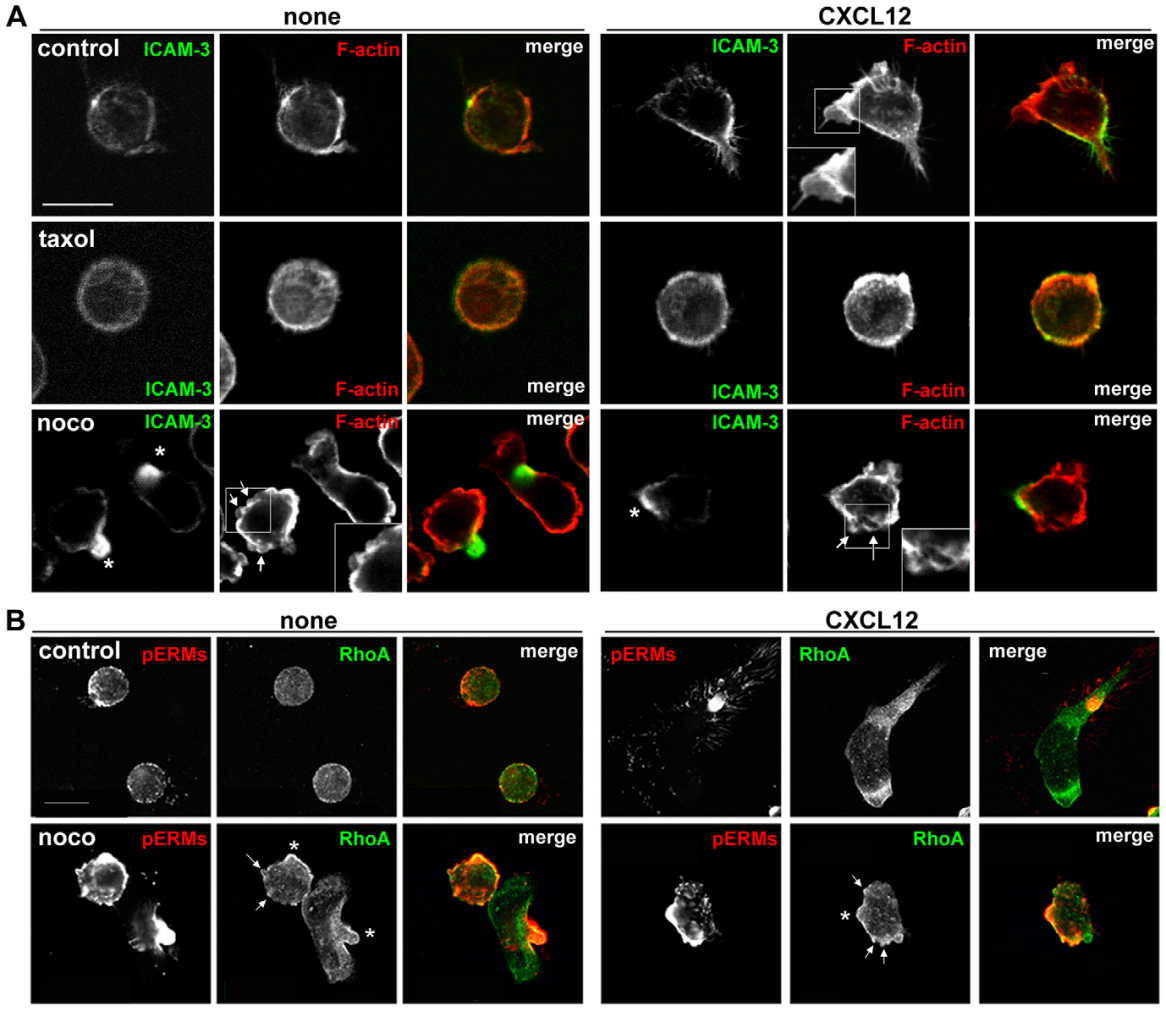
Microtubule-disrupting agents do not affect polarized clustering of uropod proteins. CCRF-CEM cells were pre-treated with or without 10 µM taxol for 30 min or 20 µM nocodazole for 10 min, plated on ICAM-1, and then stimulated with 20 nM CXCL12 for 5 min before fixation. (A) Cells were stained with anti-ICAM-3 antibody (green) and phalloidin to show actin filaments (red). Small uropod-like protrusions where ICAM-3 accumulates in nocodazole-treated cells are indicated with white asterisks. Representative confocal images are shown. (B) Cells were stained with anti-RhoA antibody (green) and anti-phospho-ERM antibody (red). Bleb-like membrane protrusions are indicated with white arrows and small uropod-like protrusions where brighter phospho-ERM staining is observed are shown with white asterisks in nocodazole-treated cells. Bar = 10 µm.

When leukocytes start to polarize, uropod-associated proteins first cluster on the plasma membrane prior to uropod protrusion [34]. The effect of nocodazole on cell polarity was investigated further by studying the localization of uropod-enriched proteins, including ICAM-3 [35], [36] and C-terminally phosphorylated ezrin-radixin-moesin proteins (phospho-ERMs) [15], [35], [37], [38]. In the majority of resting control cells, the distribution of ICAM-3 (Figure 4A) and phospho-ERMs (Figure 4B) was not polarized, whereas following CXCL12 stimulation, ICAM-3 and phospho-ERMs were predominantly localized in the uropod (Figure 4A, B). CXCL12 stimulation induced diametric distribution of F-actin and ICAM-3 to the leading edge and uropod respectively (Figure 4A). Although migratory polarization was impaired by taxol and nocodazole (Figure 1 and 2), in approximately 80% of nocodazole-treated cells ICAM-3 was concentrated in one small patch of the membrane regardless of CXCL12 stimulation (Figure 4A). Some cells had a small ICAM-3-containing uropod (Figure 4A, asterisks). Similar focal accumulation of the uropod marker CD44 [35], [39] was also observed in nocodazole-treated cells (Figure S1A, asterisk). In addition, the strongest staining of phospho-ERMs was often observed in a small patch or uropod-like protrusion (Figure 4B, asterisks). RhoA localization on the plasma membrane was higher in these patches of phospho-ERM accumulation in nocodazole-treated cells (Figure 4B), consistent with a link between RhoA and ERM phosphorylation [15], [38]. Interestingly, RhoA localized at the plasma membrane in both the uropod and lamellipodium of polarized untreated cells (Figure 4B: control+CXCL12), suggesting it could function in both locations.

Phospho-ERMs also localized on bleb-like protrusions (Figure 4B, arrows), in concordance with the observation that ezrin gets recruited to retracting blebs [32]. ICAM-3 and CD44 were not localized in these bleb-like protrusions, ruling out the possibility that they are small uropods (Figure 4A, Figure S1A, arrows). MT depolymerization therefore does not prevent polarized accumulation of uropod proteins. Despite this, nocodazole-treated cells could not form a stable uropod.

### Microtubules Regulate RhoA and ROCK Activity in T Cells

**Figure 5:**
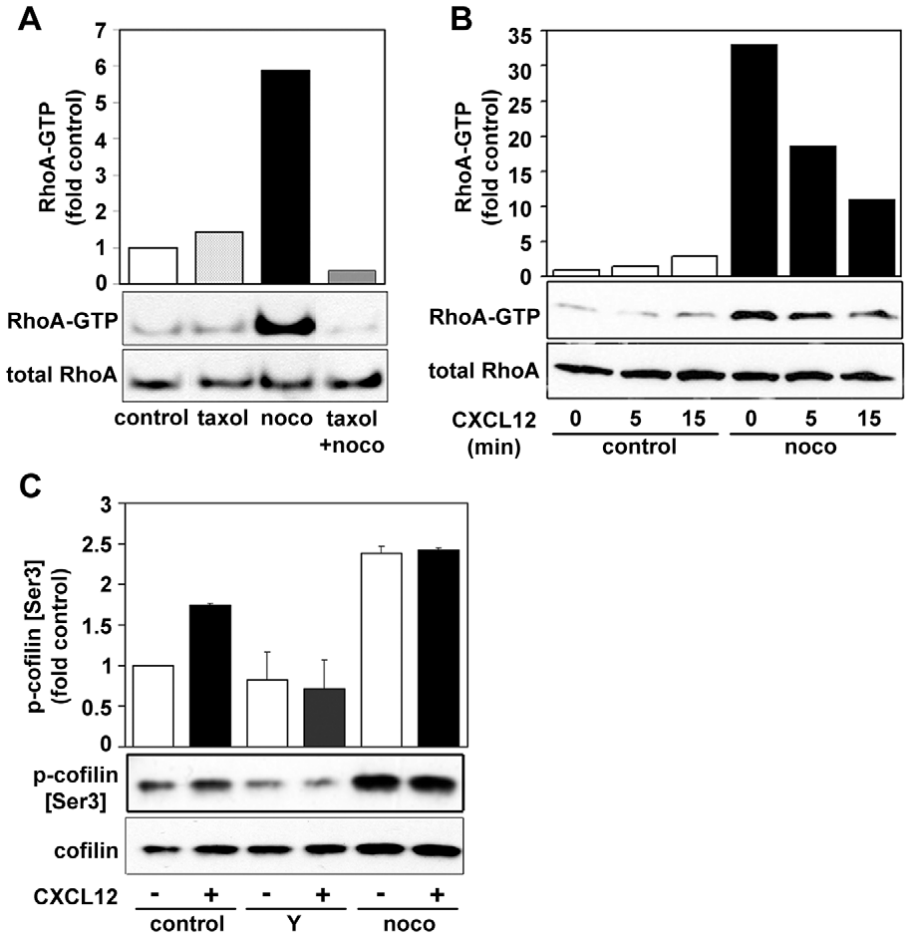
Microtubule depolymerization induces RhoA activation. (A) Cells were pretreated with or without 10 µM taxol for 30 min and subsequently with or without 20 µM nocodazole (noco) for 10 min, plated on ICAM-1 for 5 min, then lysed to determine the levels of active GTP-bound RhoA by a GST-Rhotekin-RBD pulldown assay. The graph represents a quantification of densitometry results, normalised to total RhoA and indicated as fold increase in RhoA-GTP over the level in control cells. Data shown are representative of 3 independent experiments. (B) CCRF-CEM cells were pre-treated with (black) or without (white) 20 µM nocodazole (noco) for 10 min, plated on ICAM-1, then stimulated with 50 nM CXCL12 for the indicated time periods. Levels of active GTP-bound RhoA were determined by a GST-Rhotekin-RBD pulldown assay. The graph represents a quantification of densitometry results, normalised to total RhoA and indicated as fold increase in RhoA-GTP over the level in resting control cells (0 min). Data shown are representative of 4 independent experiments. (C) Western blot of phospho-cofilin and total cofilin. CCRF-CEM cells were treated with 10 µM Y-27632 (Y) for 30 min or 20 µM nocodazole (noco) for 10 min, plated on ICAM-1 then stimulated with 50 nM CXCL12 for 5 min, lysed and analysed by western blotting using anti-phospho-cofilin (Ser3) antibody and cofilin antibody. Data represent quantification of densitometry results from 3 independent experiments (Mean ± SD), normalised to total cofilin and indicated as fold increase in phospho-cofilin over the level in untreated control cells.

Nocodazole has previously been reported to induce an increase in RhoA activity in several cell types [40]. Similarly, RhoA activity was strongly increased in CCRF-CEM cells by 20 µM nocodazole (Figure 5A). To determine whether MT depolymerization directly regulates RhoA activity, cells were treated with taxol alone or with taxol before nocodazole. Taxol alone did not affect RhoA activity, but it completely blocked nocodazole-induced RhoA activation (Figure 5A). MT depolymerization therefore triggers RhoA activation in T cells.

CXCL12 stimulation of control T cells on ICAM-1 induced a small but reproducible increase in active GTP-bound RhoA at between 15 and 30 min (Figure 5B), similar to observations in Peer T cells, PBL and Jurkat cells [30], [41]. Interestingly, the high RhoA activity in nocodazole-treated cells was slightly decreased upon CXCL12 stimulation, possibly reflecting an inhibitory effect of Rac (which is activated by CXCL12 [42]) on RhoA [43], [44].

RhoA activates the serine/threonine kinases ROCK1 and ROCK2 [16]. One of the targets of ROCKs is LIMK, which phosphorylates and inactivates the actin-depolymerizing factor cofilin. Cofilin phosphorylation was increased by CXCL12 or by nocodazole (Figure 5C), correlating with the increases in RhoA activity, and was reduced by an inhibitor of ROCK1 and ROCK2, Y-27632 [45], consistent with cofilin being a downstream target of ROCK [46].

### ROCK Inhibition Restores Migratory Persistence and Chemotaxis to Nocodazole-Treated T Cells

**Figure 6:**
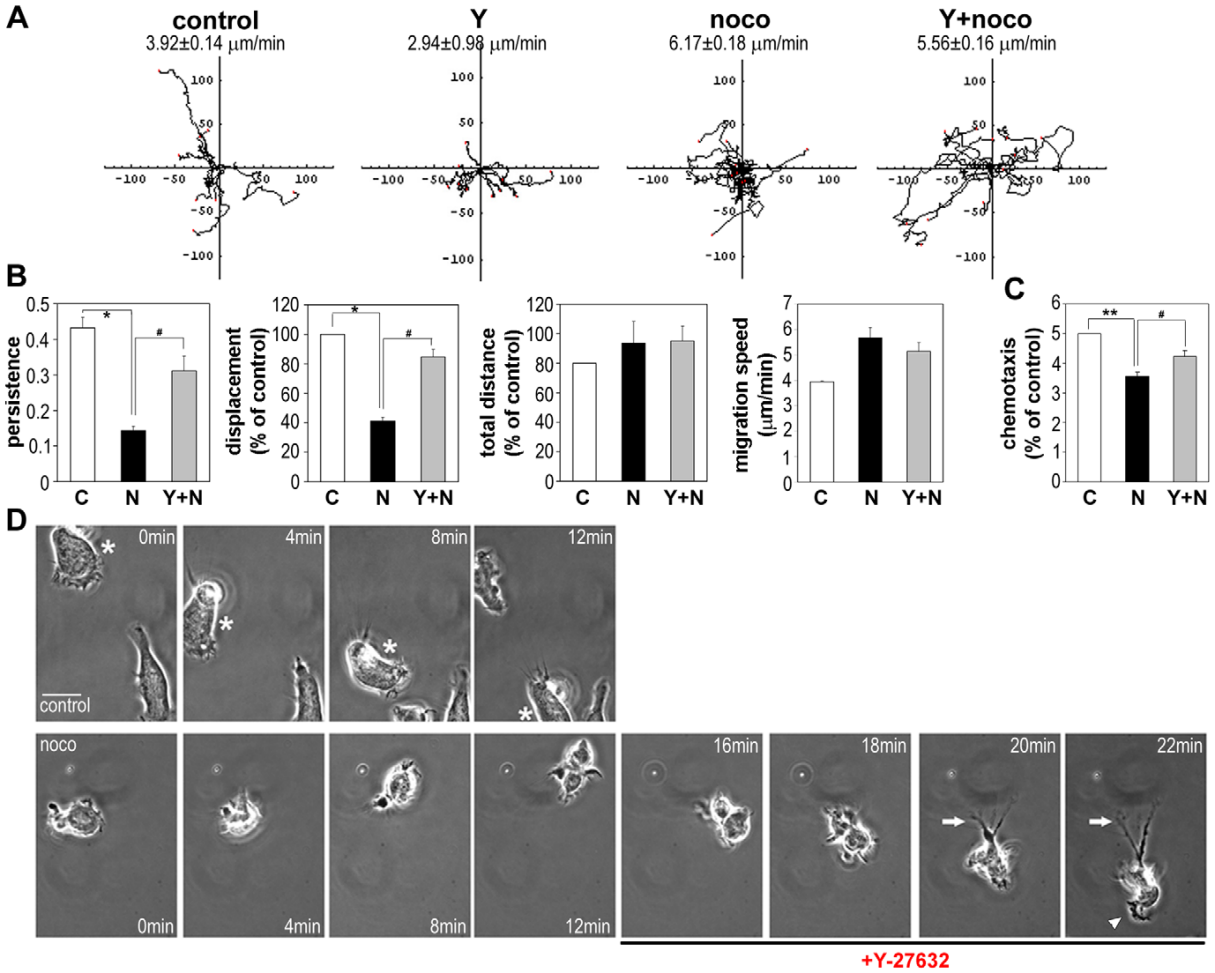
The effect of nocodazole on T-cell migration is rescued by Y-27632. CCRF-CEM T cells were treated with or without 10 µM Y-27632 for 30 min and subsequently with or without 20 µM nocodazole (noco) for 10 min, then stimulated with 1 nM CXCL12 on ICAM-1-coated dishes. (A) Cell migration was monitored by time-lapse microscopy. Examples of cell trajectories of 10 to 15 cells are shown and mean migration speed ± SEM (n = 10–15). (B) Migration parameters were calculated from cell tracks. The kinetic data of 30 to 40 cells in each condition from three independent experiments are shown. *p<0.05, compared to control cells, or ^#^p<0.05 compared to nocodazole-treated cells, indicated with bridges, Student's t-test. (C) Chemotaxis of CCRF-CEM cells towards CXCL12. Cells were pre-incubated with 10 µM Y-27632 for 30 min and/or 20 µM nocodazole for the 10 min before adding to ICAM-coated transwells. Migrated cells were counted after 60 min. **p<0.01, compared to control cells, or ^#^p<0.05 compared to nocodazole-treated cells, indicated with bridge, ANOVA. (D) CCRF-CEM cell migration was monitored by time-lapse microscopy. Y-27632 (10 µM) was added to nocodazole-treated cells (20 µM) (noco) at 12 min (Movie S9; bottom panels). A control migrating cell is marked with an asterisk (top panels). White arrow indicates a tail induced following Y-27632 addition; white arrowhead indicates restored lamellipodium. Bar = 10 µm.

The observation that nocodazole activates the RhoA/ROCK signaling pathway led us to examine whether inhibition of ROCKs could affect the migratory behaviour of nocodazole-treated cells. Cells pre-treated with Y-27632 before nocodazole addition migrated more persistently than cells treated only with nocodazole, and their displacement (start-end distance) was increased (Figure 6A, B), although total distance moved (length of track) was not affected. Y-27632 alone caused a small reduction in migration speed and cell trajectories were straighter than those of control cells (Figure 6A). In contrast, Y-27632 did not affect the migration speed of nocodazole-treated cells significantly (Figure 6A).

Consistent with our observation that nocodazole reduces migratory persistence, nocodazole decreased chemotaxis of CCRF-CEM cells towards CXCL12 in transwell assays (Figure 6C). This inhibition of chemotaxis by nocodazole was rescued by co-treatment with Y-27632, in agreement with its ability to rescue migratory persistence as determined from timelapse movies (Figure 6B).

### ROCK Inhibition Prevents Blebbing and Restores Migratory Polarity in Nocodazole-Treated Cells

ROCK has been reported to localize predominantly to the uropod in polarized human T cells [3], and Y-27632 induced elongated protrusions at the uropod enriched in MTs [30]. We therefore speculated that Y-27632 might restore T cell migration by rescuing a stable uropod structure in nocodazole-treated cells. Addition of Y-27632 during the acquisition of timelapse movies inhibited membrane blebbing and successfully re-established stable uropods in nocodazole-treated cells, allowing cells to move persistently in one direction (Figure 6D, indicated with white arrows, Movies S8 and S9). In addition, Y-27632 restored lamellipodial ruffling at the leading edge (Figure 6D, indicated with white arrowhead, and Movie S9). As previously reported for human PBLs, eosinophils and monocytes [3], [47], [48], the uropod often became elongated in Y-27632-treated cells into a long tail that remained attached to the substratum while the cell body with a ruffling leading edge continued to move forward (Figure 6D from 20 min to 22 min in nocodazole-treated cells indicated with white arrows, and Movies S9, S11, S12). However, the tail eventually retracted in most cases, allowing the cell to move persistently (Movies S10, S11, S12). Similarly, Y-27632 restored lamellipodial/uropod migratory polarity to colchicine-treated cells (data not shown). Together, these data indicate that inhibition of ROCKs rescues the polarity and migration of T cells treated with MT depolymerizing agents.

### Microtubules and ROCKs Have Similar Effects on Endothelial Migration

**Figure 7:**
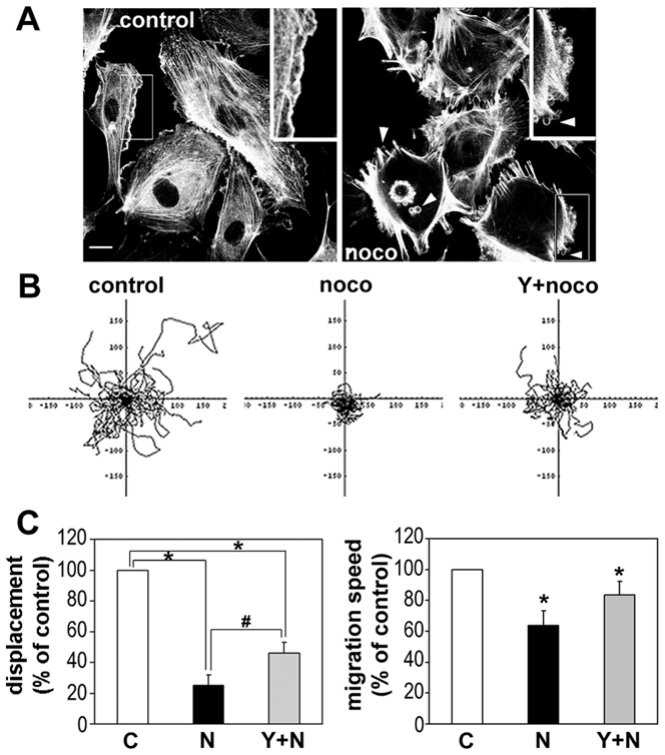
Y-27632 rescues the effect of nocodazole on endothelial cell migration. (A) Nocodazole induces membrane blebbing in HUVECs. Nocodazole (noco, 0.2 µM) was added to HUVECs for 30 min, then cells were fixed and stained with phalloidin to show actin filaments. (B) Y-27632 rescues the effect of nocodazole on HUVEC migration. Y-27632 (Y, 5 µM) and 0.2 µM nocodazole (noco) were added to HUVECs 15 min before the acquisition of time-lapse movies. The migration tracks of 90 to 100 cells from three independent experiments were analysed. Examples of cell trajectories of 30 cells in each condition are shown. (C) Migration parameters for HUVECs were calculated from cell tracks; % of control displacement and migration speed were evaluated from cells that had migrated a distance of 50 µm or more from the starting point during 5 h in (B). *p<0.05, compared to control cells, or ^#^p<0.05 compared to nocodazole-treated cells, indicated with bridges, Student's t-test.

To determine whether the effects of MT depolymerization and ROCK inhibition on cell migration were similar in a different cell type, we studied endothelial cells. MT depolymerizing agents have previously been shown to stimulate membrane blebbing in endothelial cells [49], but their effect on migration has not been characterized. Human umbilical vein endothelial cells (HUVECs) appear to be much more dependent on MTs for their migration than T cells and neutrophils, since doses higher than 1 µM induced membrane blebbing and completely abrogated cell migration within one hour (data not shown). Lower doses of nocodazole (0.2 µM) also induced membrane blebbing and increased cell contraction (Figure 7A), but only partially inhibited cell movement yet reduced cell displacement by 3.5 times as compared with untreated control cells (Figure 7B,C). Time-lapse movies showed that, like T cells, nocodazole-treated HUVECs frequently changed direction (data not shown), accounting for the lower cell displacement. Y-27632 partially rescued the defective migration of nocodazole-treated HUVECs, resulting in longer cell trajectories and a significant increase in cell displacement (Figure 7C). Since the speed of cell migration was not significantly different between nocodazole- and Y-27632/nocodazole-treated HUVECs (Figure 7C), the recovery in cell displacement was caused by a reduced turning frequency rather than an increase in migration speed.

### ROCK Inhibition Restores Cell Polarity in Nocodazole-Treated T Cells

**Figure 8:**
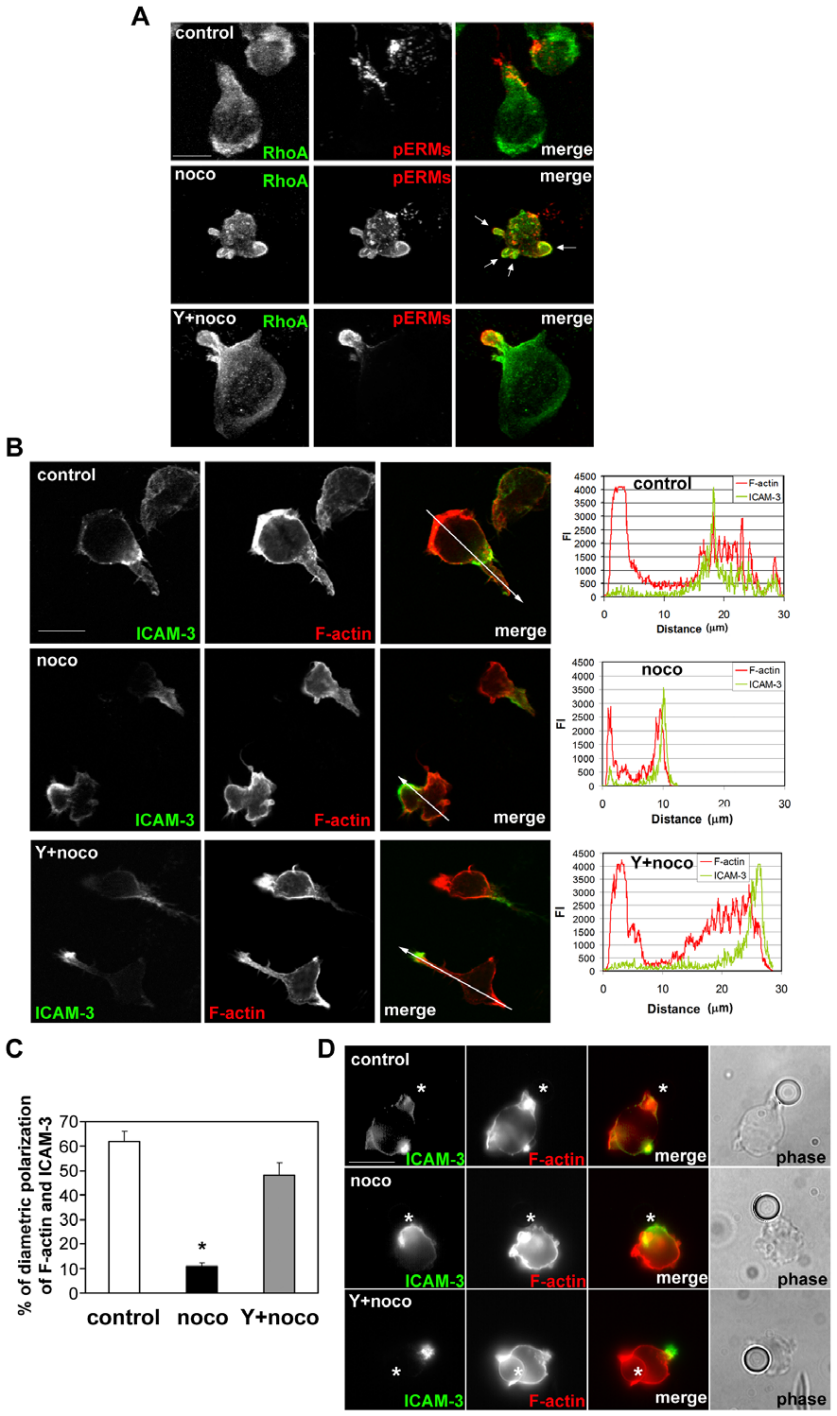
Y-27632 restores migratory polarity to nocodazole-treated cells. (A) CCRF-CEM cells were treated with or without 10 µM Y-27632 (Y) and 20 µM nocodazole (noco) then plated on ICAM-1 and stimulated with 20 nM CXCL12 for 5 min. Cells were fixed and stained with anti-RhoA antibody (green) and anti-phospho-ERM antibody (red). Representative confocal projection images reconstructed from z-stacks of 15 to 25 frames with 0.4 µm interval are shown. Note that Y-27632 prevents nocodazole-induced membrane blebbing (indicated with white arrows) and restricts phospho-ERMs to the uropod. Bar = 10 µm. (B) Localization of F-actin (red) and ICAM-3 (green) in CXCL12-stimulated CCRF-CEM cells on ICAM-1. Cells were pre-treated with 20 µM nocodazole (noco) and 10 µM Y-27632 (Y) as indicated. Representative confocal images and line-plot graphs of subcellular distributions of F-actin and ICAM-3 in each condition. Line-plot graphs indicate fluorescent intensity (FI) of phalloidin (F-actin) (red) and ICAM-3 (green) along the white arrows indicated in merge images. (C) Percentage of CCRF-CEM cells with diametric polarization of F-actin and ICAM-3. Data are from n = 220 to 250 cells in each condition which are collected from 3 independent experiments and represent mean ± SD. *p<0.03, compared to control cells, Student's t-test. (D) CCRF-CEM cells were treated with or without 10 µM Y-27632 (Y) for 30 min and subsequently with or without 20 µM nocodazole (noco) for 10 min, incubated with latex-beads coated with ICAM-1 (5 µg/ml) and CXCL12 (20 nM), and then fixed and stained with phalloidin to show actin filaments (red) and anti-ICAM-3 antibody (green). Representative images are shown. Asterisks indicate the bead-cell contact sites.

We next examined the effect of Y-27632 on cell polarization by analysing the localization of F-actin, RhoA, phospho-ERMs, and ICAM-3. As described above, most nocodazole-treated cells were unable to form uropods and lamellipodia, and instead showed multiple bleb-like protrusions where RhoA and phospho-ERMs were enriched (Figure 8A, arrows). In contrast, cells treated with Y-27632 and nocodazole formed uropods and lamellipodia with polarized localization of phospho-ERMs at uropods in response to CXCL12 stimulation (Figure 8A). Similarly, Y-27632 restored the characteristic diametric distribution of F-actin and ICAM-3 upon CXCL12 stimulation (Figure 8B, C). The same effect of Y-27632 was observed in a different cell polarization assay using ICAM-1- and CXCL12-coated latex beads: Y-27632 restored diametric distribution of F-actin and ICAM-3 in nocodazole-treated cells (Figure 8D).

### Rescue of Nocodazole Phenotype by Y-27632 Does Not Involve PI3K/Akt Signaling

Since nocodazole inhibits the establishment of a stable leading edge in T cells, and phosphoinositide 3-kinase (PI3K) activity is enriched at the leading edge of a variety of cell types including neutrophils [8], we determined whether PI3K activity was altered in T cells treated with nocodazole. A read-out for PI3K activity is Akt phosphorylation. Nocodazole decreased phospho-Akt levels in CCRF-CEM cells (Figure S1B and C), as reported in neutrophils [20]. In contrast, there was a marked increase in phospho-Akt levels in Y-27632-treated cells. However, although Y-27632 increased F-actin-rich lamellipodia in nocodazole-treated cells (Figure 8B), it was not able to restore the levels of phospho-Akt (Figure S1B and C), and thus the mechanism for ROCK inhibitor-mediated restoration of a leading edge in nocodazole-treated cells does not reflect increased activity of the PI3K/Akt pathway.

### ROCK Inhibition Stabilizes Microtubules

**Figure 9:**
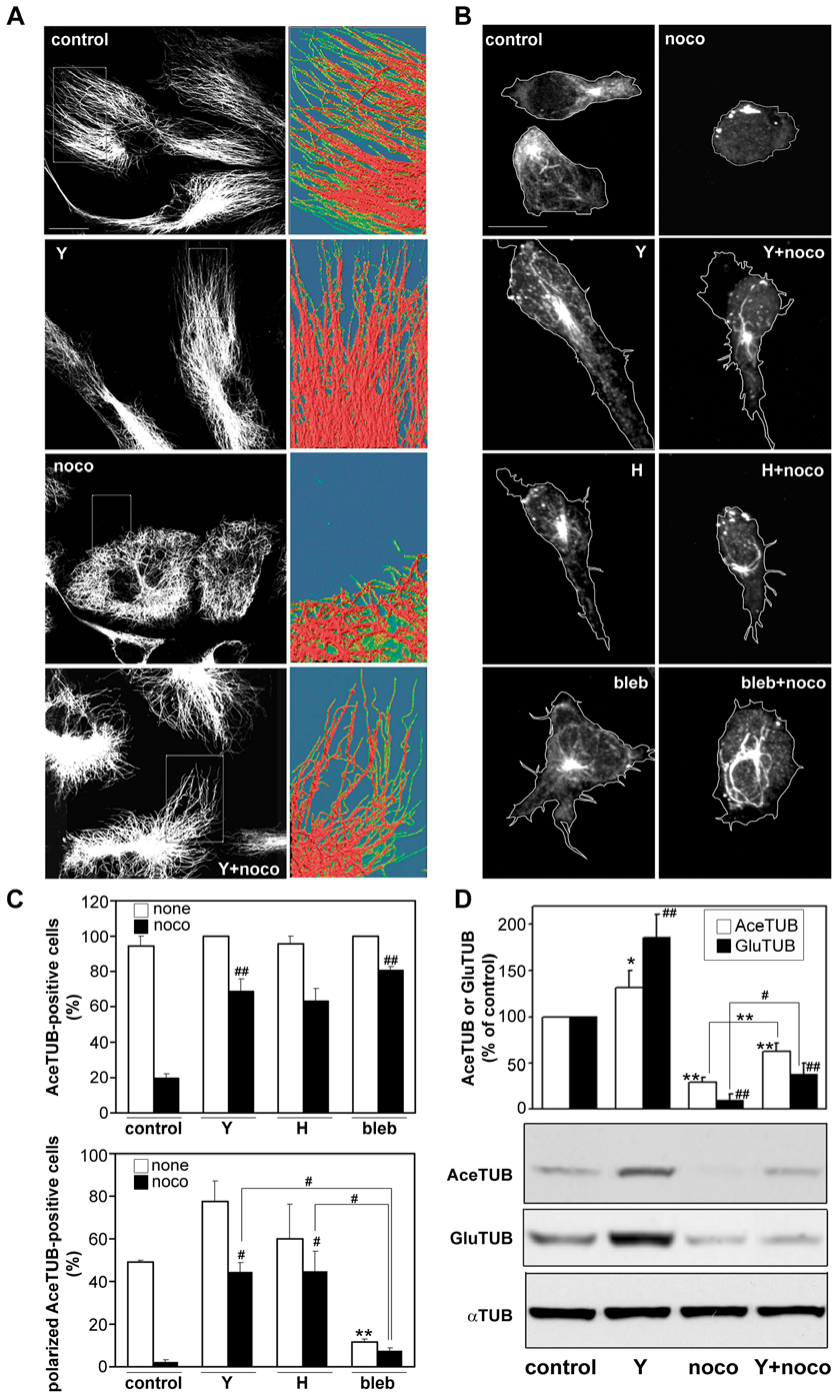
ROCK inhibitors increase microtubule stability. (A) HUVECs were left untreated (control) or were incubated with 0.1 µM nocodazole for 60 min (noco) and/or 5 µM Y-27632 (Y) for 15 min then 0.1 µM nocodazole for 60 min (Y+noco). To visualize MT distribution, cells were extracted with 0.05% Triton X-100 to remove monomeric tubulin, then stained with anti-α-tubulin antibodies. The areas marked with a white rectangle have been enlarged and pseudocoloured using the image analysis program LaserPix to help visualise MT morphology in untreated and treated cells. (B, C) CCRF-CEM cells were treated with or without 10 µM Y-27632 (Y), 0.4 µM H-1152 (H) or 50 µM blebbistatin (bleb) for 30 min, then with or without 20 µM nocodazole (noco) for 10 min, then plated on ICAM-1 and stimulated with 20 nM CXCL12 for 5 min. Localization of acetylated tubulin (Ace-TUB) was examined by staining with anti-Ace-TUB antibody. (B) Representative confocal images; the outline of cells is indicated by white lines; (C) percentage of filamentous Ace-TUB positive cells (top), and % of morphologically polarized cells (with uropod and a leading edge) containing filamentous Ace-TUB (bottom). Data represent mean ± SEM from 3 independent experiments, n = 150 to 250 cells in each condition, ^##^p<0.01 for % of Ace-TUB positive cells compared to nocodazole-treated cells in the top panel, **p<0.01 for % of morphologically polarized cells compared to untreated control, or ^#^p<0.05 compared to nocodazole-treated cells, to Y-27632+nocodazole-treated cells or to H-1152+nocodazole-treated cells (indicated with bridge), Student's t-test. (D) Western blot analysis of Ace-TUB and Glu-TUB levels in CCRF-CEM T cell lysates treated with or without 10 µM Y-27632 (Y) for 30 min and subsequently with or without 20 µM nocodazole (noco) for 10 min. Data represent quantification of densitometry results (Mean ± SD) of three independent experiments, normalised to the total α-TUB level and indicated as fold increase in Ace-TUB or Glu-TUB over the level in control cells; *p<0.05, **p<0.01 for Ace-TUB, or ^#^p<0.05, ^#^p<0.01 for Glu-TUB compared to control (without bridge), or to nocodazole-treated cells (indicated with bridge); Student's t-test.

We assessed whether inhibition of ROCKs affects MT distribution or stability, which could explain the protective effect of Y-27632 on nocodazole-induced alterations in polarization and migration. In HUVECs, low levels of nocodazole treatment for 60 min induced depolymerization of most MTs, leaving only ‘curly’ MTs around the nucleus (Figure 9A), which normally represent more stable MTs [50], [51]. Y-27632 alone did not affect overall MT distribution. However, in HUVECs treated with Y-27632 before addition of nocodazole, MTs appeared straighter and longer (Figure 9A), suggesting that Y-27632 reduces nocodazole-induced depolymerization of MTs.

In T cells, the level of stable MTs was investigated using antibodies to acetylated tubulin and detyrosinated (Glu) tubulin, which are indicators of stabilized MTs [26], [52], [53]. Over 50% of CXCL12-stimulated T cells showed morphological polarization in which acetylated MTs were concentrated in the uropod (Figure 9B, C). Nocodazole treatment almost completely eliminated acetylated MTs but approximately 40% of cells pre-treated with Y-27632 prior to nocodazole retained acetylated MTs (Figure 9B, C). Y-27632 also increased the total level of acetylated tubulin and Glu-tubulin, both in control and nocadazole-treated cells, as assessed by western blotting (Figure 9D). Since ROCKs are known to stimulate MLC phosphorylation and hence myosin II activity, we tested whether the myosin II inhibitor blebbistatin [54] affected MTs. Interestingly, blebbistatin increased the level of acetylated MTs in nocodazole-treated T cells similar to Y-27632, although it did not rescue morphological polarity in T cells (Figure 9B,C) or migration of nocodazole-treated HUVECs (data not shown). Instead, T cells treated with blebbistatin and nocodazole either had several protrusions in multiple directions or a spherical morphology (Figure 9B; data not shown). This lack of polarity could reflect a requirement for myosin II activity for stable lamellipodial protrusion [55].

## Discussion

**Figure 10:**
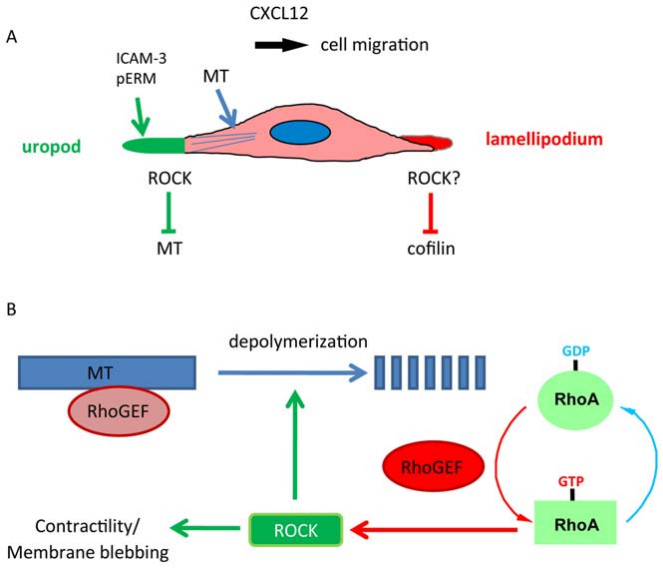
Model for roles of microtubules and ROCKs in T-cell polarity and migration. (A) T cell migration is stimulated by CXCL12. Migrating T cells have a lamellipodium at the front and uropod with uropod-localized proteins (e.g. ICAM-3, phosphorylated ERM proteins) at the back. Our results suggest that ROCKs reduces MT stability in the uropod and could inhibit cofilin activity in the lamellipodium. (B) Microtubule (MT) depolymerization, for example by nocodazole, releases a RhoGEF for RhoA, which increases the level of GTP-bound active RhoA. RhoA then stimulates ROCKs, which stimulate contractility and membrane blebbing and decreases microtubule stability. Inhibition of ROCKs reduces blebbing, increases stable MT, and reduces cofilin phosphorylation, thereby restoring lamellipodial/uropod polarity.

MT depolymerization has been shown to reduce directional migration in several cell types including human neutrophils and zebrafish macrophages [19], [21], [56]. Here, we show that MT depolymerization converts T cells from a lamellipodial/uropod migratory phenotype to a blebbing migratory phenotype, correlating with increased RhoA/ROCK activity. ROCK inhibitors prevent blebbing and restore lamellipodial/uropod polarity to nocodazole-treated cells. In addition, we have found that ROCK inhibitors and the myosin inhibitor blebbistatin protect MTs against depolymerization. Our results support a model where RhoA/ROCK signaling contributes to T cell polarization and migration by regulating both contractility and MT stability (Figure 10).

MT depolymerization has previously been reported to induce bleb-based migration in neutrophils [57] and bleb-like protrusions in the KE-37 T-ALL cell line in suspension [58], [59] or in a mouse T cell line in agarose [60], but its effects on chemokine-stimulated T cell migration or migratory polarity have not previously been investigated. Our results indicate that MT depolymerization reduces persistent migration of cells by increasing RhoA/ROCK activity, which then interferes with stable migratory polarity by causing membrane blebbing at multiple areas of the cell membrane. RhoA/ROCK signaling also mediates blebbing during apoptosis [61].

Our data demonstrate that ROCK inhibition with Y-27632 restores persistent migration in nocodazole-treated CCRF-CEM cells and HUVECs, suggesting the molecular mechanisms linking MTs and RhoA/ROCK are conserved in different types of cells. Y-27632 has also been reported to repress the inhibitory effect of nocodazole on the persistent migration of neutrophil-like HL60 cells in vitro [20] and on macrophage chemotaxis towards a wound in zebrafish embryos [21]. We show for the first time that Y-27632 can act both before and after nocodazole addition to restore migratory persistence. This reflects the multiple actions of RhoA/ROCK signaling in regulating both membrane blebbing and MT stability. Myosin II inhibition with blebbistatin is well known to inhibit membrane blebbing [61], and here we show that it also affects MT stability. Myosin IIA deficiency and blebbistatin have also been reported to stabilize MTs in fibroblasts [62]. However, myosin II inhibition is not able to restore migratory polarity in nocodazole-treated T cells, and instead cells either have multiple protrusions or a spherical morphology, and uropods are rarely observed. These two phenotypes imply that myosin II activity is required for both uropod formation and lamellipodial protrusion in T cells, as previously suggested [3], [63].

Although nocodazole inhibits uropod formation in T cells, uropod proteins and RhoA still cluster on the plasma membrane. This focal accumulation of uropod markers and RhoA could occur initially through a positive feedback loop between RhoA and ERM proteins [38], and/or RhoA and mDia1 [64]. Active RhoA would lead to phosphorylation and activation of ERM proteins, which in turn bind to and cluster uropod receptors such as ICAM-3. In the presence of MTs, RhoA activation of ROCK could destabilize MTs locally at the uropod cortex, which could in turn increase RhoA activity, probably through the release of a MT-associated RhoGEF such as GEF-H1 [24]. This positive feedback loop would establish strong RhoA/ROCK signaling in the uropod but not elsewhere, allowing cells to establish a stable lamellipodium and uropod and hence migrate persistently along one axis. MT depolymerization induces high levels of RhoA/ROCK activity at multiple points of the cell cortex, shifting cells from a lamellipodium/uropod migratory polarity to a ROCK-mediated membrane blebbing morphology with low migratory persistence (Figure 10).

As well as preventing uropod formation, nocodazole inhibits the establishment of a stable leading edge. Cofilin regulates lamellipodial extension and migratory polarity in breast carcinoma cells, fibroblasts and T cells and its activity is inhibited by phosphorylation [65]–[67]. The nocodazole-induced increase in cofilin phosphorylation could therefore contribute to the loss of stable polarity in T cells. Y-27632 inhibited the increase in cofilin phosphorylation induced by nocodazole, indicating that ROCK is a major regulator of cofilin phosphorylation in T cells, presumably acting via LIMK [68]. This could contribute to the re-formation of a stable leading edge by Y-27632 in nocodazole-treated cells. Since we observe RhoA localization in both lamellipodia and uropods, ROCK might act in both locations.

RhoA has previously been reported to increase MT stability via mDia1 but not ROCKs inquiescent fibroblasts following scratch wounding [26]. Our observation that ROCK and myosin II inhibitors affect MT stability and distribution in T cells and endothelial cells suggests that ROCK signaling to MTs could be dependent on cell type and/or culture conditions. How ROCK/myosin II signal to MTs remains to be determined. It is possible they act by regulating Rac and PAK [16], [62], which are known to induce phosphorylation and hence inhibition of the MT-destabilizing protein stathmin/Op18 [29], [69], [70]. Indeed, Y-27632 treatment increases phospho-stathmin levels in CXCL12-stimulated CCRF-CEM cells (our unpublished data). Alternatively, ROCK might directly affect enzymes that mediate tubulin acetylation and detyrosination. De-acetylation of tubulin is regulated by HDAC6 [71], and RhoA has been reported to repress HDAC6 activity during Stat5-mediated transcription [72].

In summary, our data indicate that MT destabilization induces loss of migratory polarity in T cells through RhoA/ROCK-mediated disruption of lamellipodial/uropod migratory morphology, and suggest that RhoA/ROCK signaling normally contributes to migration by affecting both actomyosin contractility and MT stability.

## Materials and Methods

### Reagents

Recombinant human CXCL12/SDF-1α and ICAM-1/Fc chimera were purchased from R&D Systems (Abingdon, UK). Nocodazole, taxol (paclitaxel), FITC-conjugated anti-α-tubulin antibody (DM1A), anti-acetylated tubulin antibody (6-11B-1) and TRITC-conjugated-phalloidin were purchased from Sigma-Aldrich (St. Louis, MO, USA), anti-phospho-Akt (Thr308 and Ser473), anti-Akt, anti-phospho-ERM and anti-ERM, anti phospho-cofilin (Ser3), anti-cofilin and anti-stathmin/Op18 antibodies from Cell Signaling (Danvers, MA, USA), anti-ICAM3 antibody from Abcam (Cambridge, UK), anti-Glu-tubulin antibody from Chemicon (Chandlers Ford, UK), blebbistatin, Y-27632 and H-1152 from Calbiochem (Nottingham, UK), Alexa546-conjugated phalloidin, Alexa488, Alexa546-conjugated goat anti-rabbit IgG (H+L) and Alexa488-conjugated goat anti-mouse IgG (H+L) from Invitrogen (Paisley, UK), goat anti-rabbit IgG (H+L)-HRP and goat anti-mouse IgG (H+L)-HRP from GE Healthcare UK Limited (Bucks, UK), anti-phospho-stathmin/Op18 (Ser16) and anti-RhoA antibody (26C4) from Santa Cruz (Autogen Bioclear UK Ltd, Wiltshire, UK).

### Cell Culture

CCRF-CEM cells, an acute T lymphoblastic leukemia cell line, were purchased from ATCC (LGC Promochem, Middlesex, UK). CCRF-CEM cells were cultured in RPMI 1640 medium with 2 mM L-glutamine containing 10% fetal calf serum, 10 mM Hepes and 1 mM sodium pyruvate at 37°C in 5% CO_2_. Cells were maintained at a density of between 2×10^5^ and 2×10^6^ cells/ml and used for experiments at passage numbers less than 10. For experiments with ICAM-1, culture dishes or coverslips were coated with ICAM-1 (5 µg/ml) in phosphate-buffered saline (PBS) at 4°C overnight. Plates were washed with PBS and blocked with 2.5% BSA in PBS for 2 h at 37°C. Human T lymphocytes were prepared from single donor peripheral blood mononuclear leukocytes by treatment with 0.5% phytohemagglutinin (PHA, Sigma, Gillingham, UK) for 48 h. Cells were washed and cultured in RPMI 1640 medium containing 10% human AB serum (BioWest, Nuaille, France) and 10 U/ml interleukin-2 (IL-2, Roche Diagnostics, Mannheim, Germany). Experiments were performed after culturing the cells for 10 to 15 days. HUVECs were purchased from Biowhittaker (Wokingham, UK) and cultured in flasks coated with 10 µg/ml human fibronectin in EGM-2 medium (Biowhittaker) supplemented with 2% fetal calf serum. For motility analysis and immunostaining, HUVECs were plated at a density of 2×10^4^ cells/ml in 9 cm^2^ Nunc slide flasks (Invitrogen, Paisley,UK) on glass coverslips coated with 10 µg/ml human fibronectin.

### Time-Lapse Microscopy

CCRF-CEM cells were incubated in migration medium (PBS containing 20 mM Hepes pH 7.0 and 0.5% BSA) at 10^6^ cells/ml for 2 hours at 37°C, 5% CO_2_. Cells were then treated with or without 10 µM taxol or 10 µM Y-27632 for 30 min and subsequently treated with or without the indicated concentrations of nocodazole for 10 min. 10^6^ cells were added to ICAM-1-coated 35-mm plastic culture dishes (Nunc) or glass bottom culture dishes (MatTek). After 5 min, non-adherent cells were removed, and pre-warmed fresh migration medium with or without the same concentrations of taxol, Y-27632 or nocodazole was added. Cells were then immediately placed on a microscope stage, equipped with a heated stage and CO_2_ circulator to maintain cells at 37°C and 5% CO_2_. Where indicated, cells were stimulated with 1 nM CXCL12. HUVECs were incubated in EGM-2 medium and plated at 2×10^4^ cells/ml in 9 cm^2^ Nunc slide flasks on glass coverslips coated with 10 µg/ml human fibronectin. Cells were treated with Y-27632 (5 µM) and/or nocodazole (0.2 µM) for 15 min prior to imaging. Cell migration was monitored by time-lapse microscopy, acquiring a frame every 15 s for a period of 30 min (CCRF-CEM cells) or every 10 min for 5 h (HUVECs).

For high-resolution analysis of membrane blebbing, 1.5×10^5^ CCRF-CEM cells or T-lymphoblasts in RPMI containing 10% serum were plated onto ICAM-1-coated glass bottom dishes. After 15 min, non-adherent cells were washed off. Where indicated, nocodazole (20 µM) was added, and then cells were imaged by time-lapse microscopy, acquiring 1 frame/s for up to 10 min.

Cells were tracked with Kinetic Imaging software (Andor Technology, Belfast, UK) and the trajectories were analyzed statistically using Mathematica software (Wolfram Research Inc, Champaign, IL). To obtain reliable values for kinetic data for CCRF-CEM cells, we collected cells in each condition whose total migration distance in 30 min was between 120–200 µm. There was no significant difference in the % of cells migrating this distance with the different treatments tested (none (n = 25): 43%; nocodazole (n = 29): 39%; nocodazole +Y-27632 (n = 30): 38%). CCRF-CEM cells that migrated less than 120 µm were not included because cells that are mostly stationary can artificially reduce persistence. For HUVECs, cell displacement was evaluated by calculating the percentage of the total cells analysed that had migrated a distance of 50 µm or more from the starting point during 5 h in each experiment.

### Transwell Chemotaxis Assay

CCRF-CEM cells (2×10^5^ cells/transwell) were incubated in growth medium alone or in medium with 10 µM Y-27632 for 30 min and/or 20 µM nocodazole for 10 min before adding (in 200 µl) to ICAM-1-coated transwells (24-well; 5-µm pore size) containing growth medium and 10 µM Y-27632 and/or 20 µM nocodazole in both wells and 30 ng/ml CXCL12 in the bottom well. After 1 h, cells in the bottom well were counted with a cell counter (CASY). Each experimental condition was carried out in triplicate in each experiment.

### RhoA Activity Assays

GST-Rhotekin-RBD protein was purified from *E. coli* by resuspending the bacterial pellet in ice-cold STE buffer (10 mM Tris (pH 8.0), 150 mM NaCl, 1 mM EDTA, 1 mM PMSF), incubating with 100 µg/ml lysozyme for 15 min on ice, and then adding 5 mM DTT, 1% Tween-20 and 0.03% SDS sequentially. The lysate was clarified by centrifugation and GST-Rhotekin-RBD was purified by incubating with glutathione-sepharose 4B beads (GE Healthcare UK Limited) for 10 min at room temperature or 1 h at 4°C. Beads were washed three times with cold STE buffer.

For RhoA pulldowns, CCRF-CEM cells were pre-incubated in low-serum RPMI containing 0.1% FCS and 25 mM Hepes overnight, and 10 to 15×10^6^ cells were resuspended in 250 µl low-serum RPMI. Cells were treated with or without nocodazole and Y-27632 as described above, then added to an ICAM-1-coated 25-mm well, incubated for 5 min, then stimulated with or without 50 nM CXCL12 for the time periods indicated in the figure legend. For Figure 5A, 10 to 15×10^6^ cells of CCRF-CEM cells were directly suspended in 10% serum containing RPMI without serum starvation, then treated with or without taxol and nocodazole as described above and used for RhoA pulldown assay. Cells were immediately lysed in ice-cold lysis buffer containing 25 mM Hepes pH 7.5, 150 mM NaCl, 1% NP-40, 10 mM MgCl_2_, 1 mM EDTA, 25 mM NaF, 1 mM Na_3_VO_4_, 10 µg/ml aprotinin, 100 µM PMSF and 10% glycerol. The lysates were clarified by centrifugation. Supernatants were incubated with GST-Rhotekin-RBD beads (∼30 µg/reaction) for 1 h at 4°C and then the beads were washed 3 times with cold lysis buffer. Bound GTP-RhoA was detected by western blotting using monoclonal anti-RhoA antibody (1∶1000).

### Flow Cytometric Analysis of F-Actin Content

CCRF-CEM cells were suspended at 10^7^/ml in RPMI containing 20 mM Hepes and 0.5% BSA and incubated for 1 h at 37°C. Where indicated, cells (10^6^ cells/treatment) were incubated in medium alone or in the presence of 10 µM Y-27632 for the last 30 min and/or 20 µM nocodazole for the last 10 min. Cells were then fixed at room temperature for 10 min by adding 200 µl of 4% paraformaldehyde, washed in PBS containing 0.5% BSA (FACS buffer), then permeabilized in 0.1% Triton X-100 in PBS for 4 min. Cells were washed in FACS buffer and then incubated for 30 min at room temperature in 100 µl of Alexa488-labelled phalloidin (2 µg/ml; Molecular Probes) diluted in FACS buffer. Cells were then washed twice in FACS buffer. 10,000 events were acquired per sample on a Becton Dickinson FACSCalibur and analysed using Cell Quest software. Data are expressed as a percentage of the mean fluorescence of untreated cells.

### Immunofluorescence

CCRF-CEM cells were incubated in serum-free RPMI containing 25 mM Hepes at 2×10^6^ cells/ml for 3 h at 3°C, 5% CO_2_. Cells were then pre-treated with or without 10 µM taxol, 20 µM nocodazole or 10 µM Y-27632 as described above, then plated on ICAM-1-coated coverslips or Lab-Tek slide chambers. After 5 min, cells were stimulated with or without 20 nM CXCL12 for 5 min. To analyse T cell polarization, cells were fixed with 4% paraformaldehyde for 10 min. For total RhoA or phospho-ERM localization, cells were fixed with 10% trichloroacetic acid for 15 min on ice. For Acetylated tubulin or Glu-tubulin staining, cells were fixed with pre-warmed 4% formaldehyde in PBS containing 2 mM MgCl_2_ and 1 mM EGTA at 37°C for 10 min, then treated with cold methanol at −20°C for 5 min. Cells were permeabilized with 0.1% Triton X-100 for 4 min and blocked with 1% BSA in PBS for 1 h at room temperature. Cells were incubated with 1∶400 Alexa546- or TRITC-labeled phalloidin, FITC-conjugated anti-α-tubulin (1∶300), anti-acetylated tubulin (1∶200), anti-Glu tubulin (1∶200), anti-ICAM-3 (1∶50), anti-RhoA (26C4) (1∶50) and/or anti-phospho-ERM (1∶100) antibodies for 1 h then with FITC- or TRITC-labeled mouse or rabbit IgG antibodies for 1 h. Images were acquired with a Photonic Science cooled CCD camera (Robertsbridge, UK) mounted on a Zeiss Axiophot microscope equipped with a 100x oil objective using ImagePro software or a LSM510 confocal microscope (Zeiss, Oberkochen, Germany) with a 40x oil objective.

To visualize stable MT in HUVECs, cells were pre-extracted to remove unpolymerized tubulin using a method modified from Gundersen et al. [73]. Briefly, HUVECs were rapidly washed in microtubule stabilizing buffer (MSB) (85 mM Pipes, pH 6.93, 1 mM EG'I'A, 1 mM MgCl_2_, 2 M glycerol, and protease inhibitors: 10 µg/ml aprotinin, 0.5 mM benzamidine, 5 µg/ml o-phenanthroline, and 0.2 mM PMSF) and then extracted with MSB containing 0.05% Triton X-100. After 2 min, cells were gently washed in MSB and fixed with methanol at −20°C. The cells were then washed in PBS, blocked with 1% BSA in PBS for 1 h then incubated with mouse anti-α-tubulin antibodies (Sigma-Aldrich), followed by TRITC-labeled goat anti-mouse antibodies. Cells were mounted in Vectashield mounting medium (Vector Laboratories Inc, CA) and examined by confocal microscopy (Bio-Rad Radiance 2100) with Bio-Rad software (LaserSharp 2000 version 5.1). LaserPix software (Bio-Rad) was used to pseudocolor MTs in images.

### Western Blotting

CCRF-CEM cells (10^7^ cells/condition) were incubated in low-serum RPMI for 3 h at 37°C, 5% CO_2_. Cells were treated with 10 µM Y-27632 and/or 20 µM nocodazole as indicated. Cells were plated on ICAM-1-coated dishes and incubated for 10 min. Cells were then stimulated with 50 nM CXCL12 for 10 min, and lysed in 100 µl of PBS containing 1% SDS. Subsequently 500 µl of PBS containing 1% Triton-X100, 25 mM NaF, 1 mM Na_3_VO_4_, 10 mg/ml aprotinin and 100 µM PMSF was added, and lysates were incubated on ice for 10 min, homogenized with a 25-gauge needle, and clarified by centrifugation. To detect acetylated and detyrosinated (Glu-) tubulin, cells were lysed as above except that pre-warmed (37°C) MT stabilisation buffer (85 mM PIPES pH 6.9, 1 mM EGTA, 1 mM MgCl_2_ and 2 M Glycerol) was used instead of PBS and lysates were not incubated on ice. Samples were analysed by SDS-PAGE followed by western blotting.

## Supporting Information

Figure S1 (A) F-actin (green) and CD44 (red) localization in control (left panel) and nocodazole-treated (right panel; 20 µM, 10 min) CCRF-CEM cells stimulated with 20 nM CXCL12 for 5 min. Asterisk indicates CD44 clustering in a small uropod-like protrusion, arrowhead indicates a bleb-like structure that does not contain CD44. (B, C) Western blots of phospho-Akt (Thr308) and (Ser473) levels. CCRF-CEM cells were pre-treated with or without 10 µM Y-27632 before addition of 20 µM nocodazole, then stimulated with 50 nM CXCL12 for 5 min on ICAM-1. The graph for phospho-Akt (Thr308) (B) represents quantification of densitometry results obtained from 3 independent experiments (Mean ± SD), normalised to total Akt and indicated as fold increase over the resting control condition. *p<0.05, compared to control CXCL12-stimulated cells, Student's t-test. The graph in (C) represents a quantification of densitometry results, normalised to total Akt and indicated as fold increase over the resting control condition. Similar data were obtained in three independent experiments.
(7.21 MB TIF)

Movie S1 CCRF-CEM T cells stimulated with 1 nM CXCL12 on ICAM-1; 1 frame/15 sec.
(2.74 MB MOV)

Movie S2 Taxol-treated (10 µM for 30 min) CCRF-CEM cells stimulated with 1 nM CXCL12 on ICAM-1; 1 frame/15 sec.
(2.47 MB MOV)

Movie S3 Nocodazole-treated (20 µM for 10 min) CCRF-CEM cells stimulated with 1 nM CXCL12 on ICAM-1; 1 frame/15 sec.
(2.48 MB MOV)

Movie S4 CCRF-CEM cell on ICAM-1; 1 frame/sec.
(2.55 MB MOV)

Movie S5 Nocodazole-treated (20 µM) CCRF-CEM cell on ICAM-1; 1 frame/sec. Note progressive membrane blebs extend in the same area of the membrane, causing forward protrusion.
(5.05 MB MOV)

Movie S6 Human T-lymphoblasts on ICAM-1; 1 frame/sec.
(4.80 MB MOV)

Movie S7 Nocodazole-treated (20 µM) human T-lymphoblasts on ICAM-1; 1 frame/sec. Note progressive membrane blebs extend in the same area of the membrane.
(1.22 MB MOV)

Movie S8 Nocodazole-treated (20 µM for 10 min) CCRF-CEM cells stimulated with 1 nM CXCL12 on ICAM-1. Y-27632 (10 µM) was added to nocodazole-treated cells at 12 min; 1 frame/15 sec. Nocodazole induces membrane blebbing, which is inhibited by the addition of Y-27632, followed by the establishment of a lamellipodium and membrane ruffling at the leading edge and a stable uropod.
(5.90 MB MOV)

Movie S9 Nocodazole-treated (20 µM for 10 min) CCRF-CEM cells stimulated with 1 nM CXCL12 on ICAM-1. Y-27632 (10 µM) was added to nocodazole-treated cells at 12 min; 1 frame/15 sec. Nocodazole induces membrane blebbing, which is inhibited by the addition of Y-27632, followed by the establishment of a lamellipodium and membrane ruffling at the leading edge and a stable uropod.
(3.27 MB MOV)

Movie S10 Nocodazole-treated (20 µM for 10 min) CCRF-CEM cells stimulated with 1 nM CXCL12 on ICAM-1; 1 frame/15 sec.
(2.20 MB MOV)

Movie S11 Y-27632- (10 µM for 30 min) and nocodazole-treated (20 µM for 10 min) CCRF-CEM cells stimulated with 1 nM CXCL12 on ICAM-1; 1 frame/15 sec. Arrows indicate examples of cells with elongated tails, which eventually detached from the ICAM-1-coated dish to move forward.
(2.09 MB MOV)

Movie S12 Y-27632-treated (10 µM for 30 min) CCRF-CEM cells stimulated with 1 nM CXCL12 on ICAM-1; 1 frame/15 sec.
(2.94 MB MOV)
