# Supplementary figures and images for: Microtubules Regulate Migratory Polarity through Rho/ROCK Signaling in T Cells
Source: PLoS One. 2010 Jan 19;5(1):e8774. doi: 10.1371/journal.pone.0008774 (PMC2808253; doi:10.1371/journal.pone.0008774)

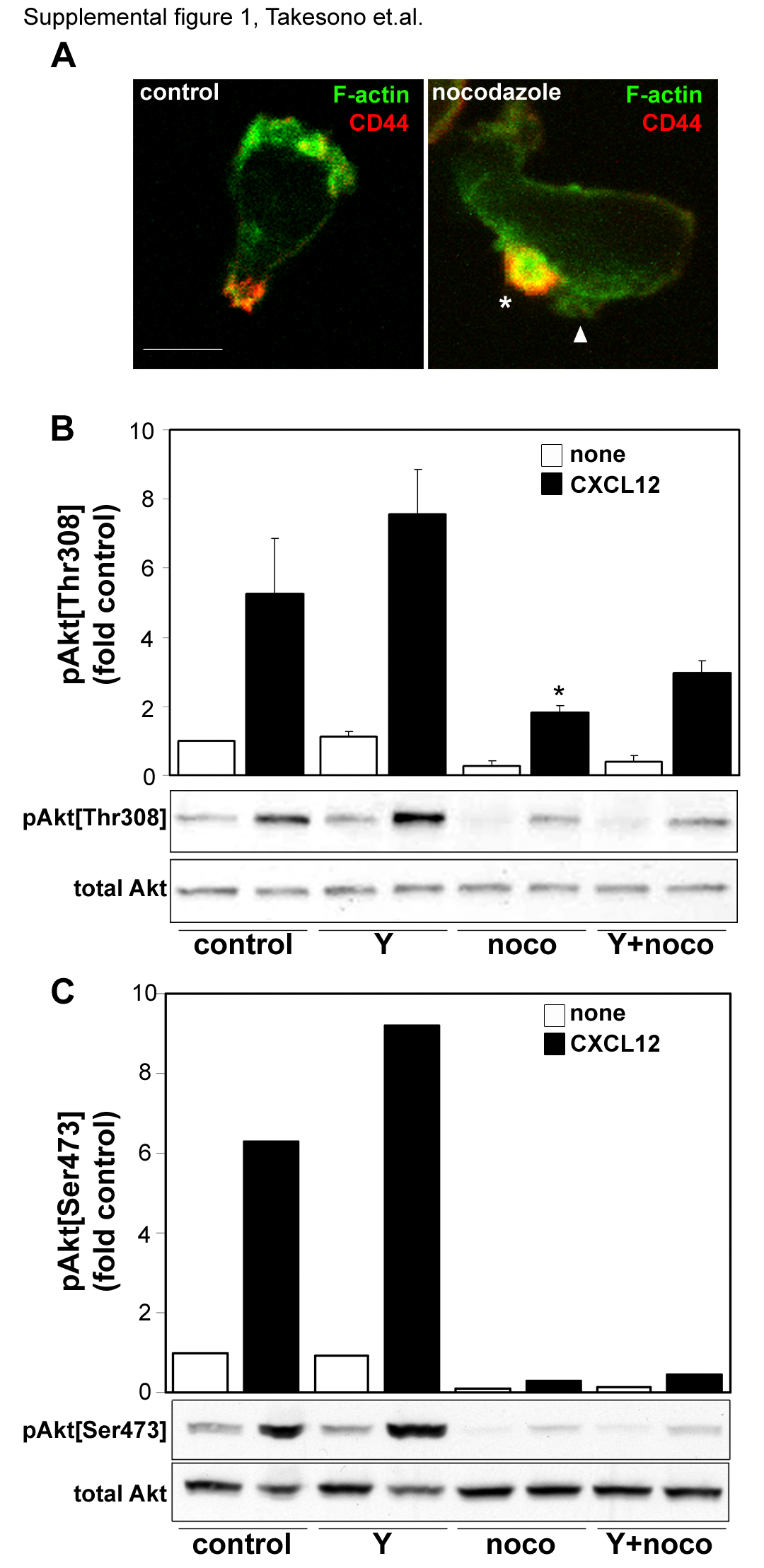

Supplement: Figure S1 — (A) F-actin (green) and CD44 (red) localization in control (left panel) and nocodazole-treated (right panel; 20 µM, 10 min) CCRF-CEM cells stimulated with 20 nM CXCL12 for 5 min. Asterisk indicates CD44 clustering in a small uropod-like protrusion, arrowhead indicates a bleb-like structure that does not contain CD44. (B, C) Western blots of phospho-Akt (Thr308) and (Ser473) levels. CCRF-CEM cells were pre-treated with or without 10 µM Y-27632 before addition of 20 µM nocodazole, then stimulated with 50 nM CXCL12 for 5 min on ICAM-1. The graph for phospho-Akt (Thr308) (B) represents quantification of densitometry results obtained from 3 independent experiments (Mean ± SD), normalised to total Akt and indicated as fold increase over the resting control condition. *p<0.05, compared to control CXCL12-stimulated cells, Student's t-test. The graph in (C) represents a quantification of densitometry results, normalised to total Akt and indicated as fold increase over the resting control condition. Similar data were obtained in three independent experiments. (7.21 MB TIF) [file pone.0008774.s001.tif]
